# Supplementary material for: Leukemic fusion genes repress viral gene expression and expel adenovirus from persistently infected human B lymphocytes but evidence of the virus lingers behind
Source: bioRxiv. 2025 Dec 23:2025.12.19.695471. Preprint. [Version 1] doi: 10.64898/2025.12.19.695471 (PMC12767664; doi:10.64898/2025.12.19.695471)
Supplement: Supplement 1 — Figure S1. Stable expression of ETV6/RUNX1 or RUNX1/MTG8 does not affect the growth of persistently infected B lymphocytic cells. A persistent infection of Ad5dl309 was established in BJAB cells stably transduced with the empty vector or expression vectors for the indicated leukemic fusion genes. At 28 days post infection, cell cultures were established at 1×10^5^ cells per ml. The number of viable cells was determined daily for 12 days without supplementing the growth medium. The population doubling time (PDT) was calculated by a log-linear regression for the values up to 10 days. Expression of the leukemic fusion transcripts was confirmed in these same cultures and shown in Fig. 1. [file media-1.pdf]

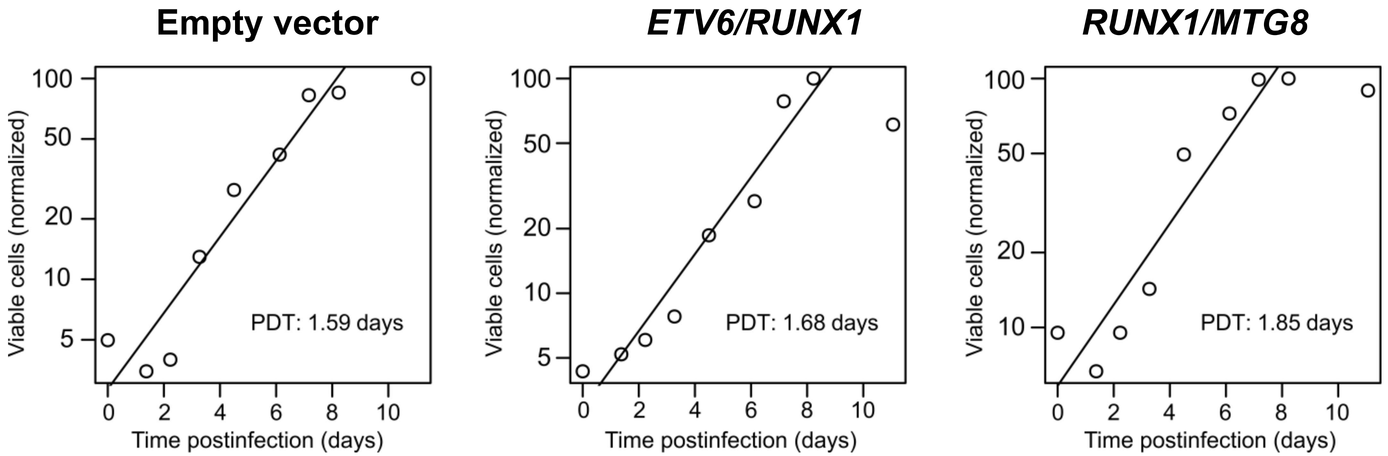


**Figure S1.** Stable expression of ETV6/RUNX1 or RUNX1/MTG8 does not affect the growth of persistently infected B lymphocytic cells.
